# Reply to “A warning against over-interpretation of seasonal signals measured by the Global Navigation Satellite System”

**DOI:** 10.1038/s41467-020-15103-4

**Published:** 2020-03-13

**Authors:** Dibyashakti Panda, Bhaskar Kundu, Vineet K. Gahalaut, Roland Bürgmann, Birendra Jha, Renuhaa Asaithambi, Rajeev Kumar Yadav, Naresh Krishna Vissa, Amit Kumar Bansal

**Affiliations:** 1 Department of Earth and Atmospheric Sciences, NIT Rourkela, Rourkela, 769008 India; 2 CSIR-National Geophysical Research Institute, Hyderabad, 500007 India; 3 Department of Earth and Planetary Science, University of California, Berkeley, CA 97720-4767 USA; 4 Department of Chemical Engineering and Materials Science, University of Southern California, Los Angeles, CA 90007-1211 USA; 5 Institute of Seismological Research, Gandhinagar, Gujarat 328009 India

**Keywords:** Geophysics, Tectonics

**Replying to** K. Chanard et al. *Nature Communications* 10.1038/s41467-020-15100-7 (2020)

Panda et al.^1^ analysed 41 continuous Global Navigation Satellite System (GNSS) sites from the Nepal and Garhwal-Kumaun region of the Himalayan arc and found that a few sites, which are located northward about 100 ± 20 km from the Main Frontal Thrust, show higher-amplitude seasonal transients in the horizontal component (predominantly in the north) of the GNSS coordinate time series. They ascribed these transients to changes in aseismic slip rate on the deep megathrust that may be controlled by seasonal hydrological loading^1^. They further invoked that modulation of aseismic slip on the megathrust down-dip of the seismogenic zone is due to the fault resonance process, induced by the seasonal hydrological stress changes.

Chanard et al.^2^ in their comments contest for some GNSS sites in our finding that the higher amplitude horizontal seasonal geodetic signal in the Garhwal-Kumaun and Nepal Himalaya is significantly larger than predicted by the global hydrological models. Furthermore, they argue that such higher amplitude horizontal seasonal transients in the GNSS time series could be due to unmodelled surface mass variations, thermo-elastic deformation of the monument and bedrock influenced by seasonal variations in surface temperature, poroelastic deformation related to variations in water table, systematic errors due to unmodelled semidiurnal (or diurnal) tides, tropospheric delay, environmental effects (e.g., snow, ice, soil moisture, vegetation growth, etc.), and draconitic errors. We agree that surface mass variations are not fully modelled in the available global hydrological load datasets and can cause spatial variation in the GNSS transients. It has also been argued that thermoelastic deformation can influence horizontal displacements up to a few millimeters at short spatial wavelengths^2^. However, we suggest that other factors, namely unmodelled semidiurnal (or diurnal) tides, tropospheric delays or draconitic errors, may not likely to cause significant variations within a small region or between adjacent GNSS sites that are located within 50 km or less^3,4^. Water table variations are the largest in the Indo-Gangetic plains and Sub-Himalaya, where such anomalous transients are absent. Therefore, we partly disagree with the arguments made by Chanard et al.^2^.

The anomalous transients reported by Panda et al.^1^ are observed at some of the GNSS sites that are located above the base of the seismogenic fault, i.e., the Main Himalayan Thrust (MHT). There is evidence that a mid-crustal ramp close to the base of the seismogenic MHT may only exist on some Himalayan sections^5,6^. Panda et al.^1^ argued that the GNSS sites above or close to the ramp may exhibit higher transient motion near regions where subsurface fluids are abundant at the MHT. Fluids at high pressure are commonly invoked to explain the slow-slip transients occurring at deeper level on continental and subduction faults around the world^7–9^. A few Magnetotelluric studies and observations of seismic swarms support the presence of fluids at the base of the seismogenic MHT^10–12^. A few earthquake swarms have been reported from Nepal Himalaya (e.g., Dharchula swarm, Sarshin swarm, Gudelhongu swarm, Hoste-Colomer et al.^13^ in their Table 1 and Fig. 6) and from Garhwal-Kumaun Himalaya (Gopeshwar swarm)^14^ which also imply the presence of fluids in these regions.

Interestingly, all four of the GNSS sites showing strong anomalous transients (i.e., DRCL, BYNA, CHLM and HRMN, reported by Panda et al.^1^) are located very close to the reported swarms. DRCL and BYNA are located close to the 1997 Dharchula swarm^13^ and a mid-crustal seismicity cluster in western Nepal captured by HiKNet^12^. CHLM is located close to the 1997 Sarshin swarm^13^, and HRMN is located close to the 2009 Gopeshwar swarm^14^. We suggest that mid-crustal swarms in the Nepal and Garhwal-Kumaun region of the Himalayan arc can be attributed to fluid-rich isolated zones and could be responsible for anomalous transients^1^. In fact, Hoste-Colomer et al.^12^ remarked bilateral migration of a seismic sequence in western Nepal (which are located exactly in the regions of the GNSS sites DRCL and BYNA), possibly induced by fluid diffusion or propagation of a local slow slip event along some sections of the MHT. Nonetheless, a direct association of seismic swarm activity and geodetically measured transient motions has not been documented yet, due to the limited existing monitoring capabilities.

The effect of the fault resonance process can also be influenced by the presence of fluids (Supplementary Fig. 9 in Panda et al.^1^). Panda et al.^1^ showed that there is a several-fold increase in the amplitude of the modelled velocity perturbation (i.e., fault resonance process) under conditions of increased pore-fluid pressure (or lowering of effective normal stress). Therefore, we posit that isolated fluid-rich regions at the base of the seismically active MHT act as conditionally stable frictional domains^15^, making them more sensitive to periodic stress perturbations by hydrological loading. Furthermore, we have observed similar correlations between GNSS sites exhibiting higher amplitude of seasonal transients and fluid rich deeper crustal regions in other orogenic belts and we hope to model the transient contribution from the fluid-filled regions in future work.

Thus we suggest that although there is uncertainty in the contribution of global surface load models towards causing transients in the GNSS time series, the fact that the anomalous transients at GNSS sites in the Himalayan region are only seen at some of the sites close to the base of seismogenic MHT hints that they could be due to transient slip on a fault ramp in the presence of crustal fluids at high pressure. Therefore, based on only the ratio of horizontal and vertical transient (as suggested by Chanard et al.^2^), we may not compare the cases of anomalous transients at GNSS sites in the Himalayan region with other GNSS observations elsewhere (as presented in Fig. 2c of Chanard et al.^2^). We agree that in the absence of modelling of high transients in the fluid rich regions, the geodetic deep slow-slip evidence presented so far remains circumstantial and more data are needed to address this process. At the same time, we are hopeful to detect such anomalous transients with more GNSS and seismic station coverage in the Himalayan arc, which may lead to better understanding of seasonal modulation of deep slow-slip on the MHT.

## Data Availability

No new data were generated for this comment.
